# From social interactions to interpersonal relationships: Influences on ultra-runners’ race experience

**DOI:** 10.1371/journal.pone.0225195

**Published:** 2019-12-02

**Authors:** Brian Harman, Céline Kosirnik, Roberta Antonini Philippe

**Affiliations:** 1 Leicester Castle Business School, De Montfort University, Leicester, United Kingdom; 2 Institut des Sciences du Sport, Faculté des Sciences Sociales et Politiques, University of Lausanne, Lausanne, Switzerland; University of Sao Paulo Medical School, BRAZIL

## Abstract

**Objectives:**

Ultra-running’s psychological and physiological dimensions have been widely studied. However, the social dimensions of ultra-racing have been largely overlooked. This study aims to identify the different types of social interactions that occur during a multi-day, ultra-race and to understand how these interactions influence ultra-runners’ race experience.

**Methods:**

Athletes competing in Britain’s “Spine Race” were recruited for the current study. Twelve male runners aged between 32 and 66 years (M = 50; SD = 10.01) followed the qualitative protocol designed for the study. Specifically, each participant completed a modified version of the Day Reconstruction Method (DRM) instrument and underwent a post-race, self-confrontation interview. Participants were asked to recall and reconstruct their memories about their (a) actions (e.g., What did you do when you encountered other people?); (b) thoughts (e.g., What were your thoughts when you encountered other people?) and (c) feelings (e.g., What were your feelings when you encountered other people?).

**Results:**

The results show that social interactions and interpersonal relationships are common in ultra-races. Different groups of people play an important role in racers’ motivation and goal achievement: fellow racers, volunteers, medics, friends and family, and even the general public. The resulting social interactions and relationships can occur before, during and after the race in both online and offline environments. These social influences have a powerful influence on the athlete’s race experience.

**Conclusion:**

The vast majority of social interactions reported by runners had a positive effect on their race experience and often led to interpersonal relationships. Ultra-runners should be mindful of the impact that social interactions and relationships can have in shaping their race experience.

## Introduction

An ultra-marathon or “ultra” is defined as any footrace that exceeds the marathon length of 42 kilometres. In recent years, there has been a rapid growth in ultra-marathons [1]. It has long been known that ultra-runners are subjected to a wide variety of stressors that create both physiological and psychological challenges [2, 3, 4, 5]. Negative emotional states [6], sleep deprivation [7] and intense fatigue [8] are just some of the well-documented challenges associated with ultra-running. In sport psychology literature, past research has explored many of the psychological traits exhibited by ultra-runners. For example, successful ultra-runners tend to score high on traits such as extraversion and experience seeking [9], emotional intelligence [10], sport confidence [11] and mental toughness [12, 13]. Ultra-runners have been found to deploy a variety of cognitive strategies to overcome the challenges they encounter [8, 14, 15, 16]. Research suggests that intrapersonal factors can determine part of an athlete’s personal response to race related stressors [17]. In contrast, the interpersonal factors that determine an athlete’s response to race related stressors are less well known. While the influence of interpersonal factors are broadly debated within the field of psychology, the influence of interpersonal factors within sports psychology are less contentious. Indeed, the influence of interpersonal factors on team performance has been well documented within the field of sports psychology [18, 19]. However, the same cannot be said of individual sports such as ultra-running. Preparing for an ultra-marathon demands high levels of training and often necessitates that the athlete trains alone [20]. Research suggests that ultra-marathoners train differently to marathon runners [1, 21]. As such, these athletes must be independently minded and willing to forgo the psychological support offered by a team during pre-race training. Correspondingly, the motivation exhibited by ultra-runners is found to differ from that of runners in shorter races and athletes within other sporting disciplines [22, 23, 24]. Although ultra-runners often adopt a more solitary approach to training, they do use online forums and discussion groups to exchange training tips and offer advice [25].

While the prospect of social interaction is minimal during pre-race training, the potential for social interactions during an ultra-marathon race is much higher. The longer distances being covered and the presence of other athletes provide fertile conditions for social interactions between fellow racers, medical staff, volunteers and the general public [26]. In short, during longer, multi-day races, the potential for social interaction increases drastically. Therefore, while ultra-runners typically train alone, it does not necessarily follow that ultra-racers maintain this independent mindset throughout an actual ultra-marathon race. Indeed, few athletes can remain completely self-sufficient throughout a long ultra-race. Anecdotal evidence suggests that athletes frequently form ad hoc groups during races. While dependent upon situational affordances and pragmatic considerations, this group formation process appears to occur organically and without the premeditated intent of athletes [27]. Forming groups is frequently a sound strategic decision as it allows the group to leverage the strengths and skills of different team members (for a review see [28]). Past research has demonstrated that group membership offers a number of important rewards (for a review see [29]). Indeed, research suggests that team membership provides athletes with emotional, informational and esteem support in addition to increased resource sharing opportunities [30]. Being in a group might also increase motivation and provide psychological support for racers during an ultra-marathon race.

Teamwork and social interaction are integral components of many sports. However, we observe that the literature has largely overlooked the social aspects of ultra-running. This is perhaps due to the fact that ultra-running is generally regarded as a singular and solitary pursuit. However, this perception is a simplification of the true picture. Athletes do not perform in a ‘social vacuum’ and future research must acknowledge this fact. Indeed, scholars have recently called for future research to investigate the role of communal coping in different contexts [28]. Communal coping refers to “a process whereby stressors are appraised and acted upon in the context of close relationships, and it describes the efforts of individuals in a group as they collectively cope with stressors” ([28], p. 507). Communal coping is therefore contingent upon social interactions and relationship formation. Relationship scholars describe an interaction as an episode with a limited span of time whereas an interpersonal relationship involves a longer duration of time during which partners influence each other’s activities [31]. The current research seeks to address the dearth of literature on the social aspects of ultra-running. We hypothesis that social interactions can affect an athlete’s race experience and their ability to cope with the race situation.

## Materials and methods

### Participants

The population was composed of twelve volunteer participants (8 Spine Fusion race athletes and 4 Spine Flare race athletes). All participants were male and aged between 32 and 66 years (M = 50; SD = 10.01). Participants all had a history of participating in ultra-marathons and were deemed eligible for the Spine race. As part of the application process, first time racers are required to submit their race CV so that they can prove they have sufficient race experience and mountain skills to safely participate in the race. In the current study, seven of the eight Spine Fusion athletes completed their race while three of the four Spine Flare athletes completed the race.

The Spine Race was chosen because it ranks among the toughest ultra-marathons in Europe. Indeed, it is widely regarded as one of the world’s toughest endurance races (see information on https://thespinerace.com). In addition, the first author was known to the race organizers. He had previously undertaken the Spine race and was familiar with the race format. Beginning in the English Lake District and finishing at the Scottish border village of Kirk Yetholm, the race route snakes its way up the remote and mountainous “Spine” of England along Britain’s famous national trail, the Pennine Way. The race route is 436 km in length and boasts a cumulative height gain of 16,230 meters. Racers, known as “Spinners”, are self-supported athletes who endeavor to complete the route within the allotted 168 hours permitted. The winter Spine Race takes place in mid-January when limited daylight and adverse weather conditions make the race especially challenging. The Spine Challenger is the “fun run” alternative to the Winter Spine. This race is 174 km in length and is held in conjunction with the Winter Spine Race.

The Spine Fusion race and the Spine Flare are recent additions to the Spine Race series. The Spine Fusion and the Spine Flare races are summer versions of the Winter Spine Race and the Spine Challenger race respectively. The participants in the current study are athletes who participated in the second year of Spine Fusion and Spine Flare races (July 2018).

### Procedure

The principal investigator obtained ethical approval for this project from the Faculty of Business and Law at De Montfort University prior to starting the study. A purposive sampling approach was adopted in the current study. Specifically, participants were recruited by posting a recruitment appeal on the official Spine Race Facebook group. The race organizers who moderate this closed Facebook group granted the lead author permission to make contact with racers through this webpage. In the recruitment appeal, the aims of the research were outlined and interested parties were encouraged to make contact if they wished to volunteer for the study. Those who responded to the recruitment appeal completed an informed consent form before they were accepted into the study. Standardized information and instructions for the questionnaires and interviews were provided to participants. Participants were reassured that (a) participation in the study was strictly voluntary, and (b) the collected data would only be used for research and would remain strictly confidential.

The study was composed of two sub studies that were designed to collect pre-race and post-race data. Six weeks before the race, participants were emailed a questionnaire in which they recorded their biographic data, training regimes, sporting backgrounds and motivations for undertaking the race. Participants also received a modified version of the Day Reconstruction Method (DRM) instrument [32]. The modified DRM was sent to participants in advance of the race to ensure they were familiar with the format of the document and the information being sought. After the race, participants received a copy of the modified DRM immediately upon finishing their race. This ensured that participants could record their race story while the details of events were still fresh in the minds. All participants completed this form in advance of the post-race interview which took place one to three days after the Spine Fusion/Spine Flare races. To ensure participants’ anonymity, they are identified under the following identifications; P01, P02, P03, etc.

### Measures

This study adopts hermeneutic phenomenology as the research approach. Hermeneutic phenomenology aims to analyze experience by describing a phenomenon in terms of how it emerges from the fringe of consciousness and how it affects individuals on a personal and social level [33, 34, 35]. In acknowledging the contextual nature of the data generated, this approach aims to achieve a sense of the meaning that others give to their own situations. Phenomenological approaches are widely used in the social sciences. Increasing, these approaches are being used by researchers to understand the psychological processes of trail running athletes [36, 37].

The modified Day Reconstruction Method (DRM) instrument is an instrument used to help respondents systematically recall and reconstruct their memories of events. The instrument prompts respondents to divide their day into distinct time periods of action known as “episodes”. Thus, the memory of a whole day is composed of a series of episodes that link to each other sequentially. The modified DRM prompted participants to document their race story in a series of episodes that described their activities and experiences at different locations on the race route. The instrument was specifically designed to help racers recall their race by prompting them to recall memories of different episodes. The DRM resembled a route card that listed geographic landmarks and race checkpoints that athletes encountered during the race. Participants were also asked to record their thoughts, feelings, actions and goals at each location (i.e., episode). Once participants had completed the DRM, they underwent a self-confrontation interview, one to three days after the race.

In the self-confrontation interviews, participants were asked to give a full account of their race from start to finish. Self-confrontation interviews are designed to collect data on a previously experienced situation. The interviewer asked the participants to re-situate, explain and re-enact specific situations at a pre-reflexive level of experience [38]. This approach accommodates the underlying assumptions of hermeneutic phenomenology by focusing on the structure of an experience and the meaning an individual assigns to it [35]. The DRM was used by the interviewer to guide the interviewees. Participants referred to their completed DRM to help them recall their race and to provide additional details when probed about specific aspects of their race experience. The interview questions focused on the athlete’s social interactions with others. Specifically, the questions focused on three aspects of social interaction (a) actions (e.g., What did you do when you encountered other people?); (b) thoughts (e.g., What were your thoughts when you encountered other people?) and (c) feelings (e.g., What were your feelings when you encountered other people?). In addition, participants were asked to describe the challenges they encountered during the race and to explain how they coped with these challenges.

These self-confrontation interviews lasted 60–120 minutes and were recorded. Five of the 12 interviews were conducted in person. The remaining 7 interviews were conducted using Skype. The large geographic spread of racers along the route meant that it was logistically impossible to conduct every interview in person.

### Data analyses

The interviews were transcribed and subsequently analyzed using NVivo V.10 software. Participant’s thoughts, emotions and actions were coded in order to identify the social interactions that helped them to cope with the challenges they encountered during the race.

The qualitative data analysis utilized a thematic analysis framework. This approach involves “a constant moving back and forward between the entire data set, the coded extracts of data that you are analyzing, and the analysis of the data that you are producing” ([39], p. 86). The analysis proceeded in a number of steps. The researchers became familiar with the data through transcription and spent some time re-reading the script and re-listening to the audio recordings. Open coding determined the separation of the data initially; answers from different sections were able to be coded under the same heading at this point. Following the guidelines from [39] initial codes were generated and line-by-line analysis was conducted in order to gather relevant data for each potential theme. The coded themes were isolated and more specific themes within each section were identified.

We employed several techniques to ensure the trustworthiness and credibility of the data. First, all three researchers were familiar with qualitative analysis. Second, we took a collaborative approach in the process of data analysis to reduce interpretive bias. The members of the research team independently identified and coded meaning units in each of the interview transcripts. The team, subsequently, engaged in several reflective discussions to ensure agreement in the process of interpretation and the coding of themes. This ensured coding reliability (inter-rater reliability) and minimized interpretive bias [40]. The process of reflection and verification between the researchers continued until all themes were agreed and verified, after which time, final themes and categories were established.

## Results

The study reveals that racers have different interactions with different groups of people during the race: 1) fellow racers, 2) race officials (i.e., medics and checkpoint volunteers), 3) family, friends and followers and 4) the general public. The interpersonal relationships formed as a result of these social interactions are the focus of the current research.

Participants reported engaging in a wide variety of social interactions during the race which strongly influenced their subjective race experience. These interactions were often based on shared situational goals that developed organically throughout the race. The vast majority of social interactions reported by participants were judged to have a favorable effect on their race experience. However, some social interactions produced undesirable outcomes.

Overall, the results suggest that ultra-running is more socially constructed than previously acknowledged in the literature. Indeed, the findings undermine the commonly held perception that ultra-running is a solitary sport. This erroneous image of solitary individualism is not a true reflection of the sport. The results provide evidence that social interactions are a fundamental component of multi-day endurance events.

The opportunities for meaningful social interactions naturally increase with race length. Accordingly, the current research found that social interactions tended to increase among racers as the race progressed. Therefore, long distance ultra-races such as the Spine race are perhaps more likely to have a stronger social dimension than shorter ultra-races. It may also be the case that the challenges faced during longer races are particularly amenable to social solutions. A summary of the social influences that affected athletes on the Spine race are summarized in **Table 1**.

**Table 1:** Social interactions and relationships influencing the runners.

Dimension	Theme	Sub-themes
Fellow racers	Influence of competitive racers	Pre-race nerves Competition stunts social interaction
	Functional teams	Psychological benefits of teamwork: time dilation, distraction from discomfort and boredom, increases feelings of safety Division of labor Problem solving options and shared goals
	Dysfunctional teams	Conflict: team goals vs. individual goals
Race officials	Influence of Medics	Social bonds through multiple interaction Fear of medics exerting expert power
	Influence of Race Volunteers	Mutual respect and a pay-it-forward ethos Facebook group
Family, friends/ followers	Direct support	Family members’ increasing emotional stability
	Indirect influence	Family members’ undermining emotional stability Support via social media
General public	Direct support	Encouragement
	Indirect pressure	Resourceless back markers Misinformation

### Fellow racers

Social interactions with other racers often occurred before the race and during the race. The results of the current study suggest that pre-race and in-race social interactions can have a detrimental effect on a racer’s mindset. This is especially true if the racer is focused on the competitive aspects of the race. Different themes are developed through this dimension: influence of competitive racers, functional teams and dysfunctional teams.

#### Influence of competitive racers

Pre-race nerves. Some participants in the current study revealed that their pre-race nerves are accentuated by the presence of other nervous racers. Indeed, one racer enacted avoidance strategies to limit his exposure to other racers before the race:

You talk to other runners and it’s all about…oh isn’t it going to be a long way, oh it’s going to hurt. Its negative stuff so his advice (race coach) was always just don’t speak to anyone—(P07)

Pre-race nerves can also lead racers to question their own decisions and gear choices. This line of internal questioning can have a detrimental effect on the athlete’s race focus. These internal questions and doubts were further exasperated when they compared themselves to other racers.

Competition stunts social interaction. The negative social influence of other racers is not confined to pre-race procrastination. Indeed, for competitive racers, the close proximity of other racers is a source of irritation throughout the race. Other racers may force a racer to deviate from their race plan and therefore provoke them into running at a pace that is often incompatible with their pre-set objectives.

I really hate having to run at someone else’s pace…. It’s always nice to feel that the other guys running at your pace because running at a pace that isn’t your own is mentally draining and that was one of my biggest fears. I needed to have the confidence that even if someone did go off faster than I did, I’d get them later. There’s that temptation to try and keep up with them–(P07)

A racer who is overtaken by another racer may also feel annoyed and frustrated for relinquishing a race position. This can have a detrimental effect on racers’ mood and can jeopardize their mindset if not carefully managed and rationalized.

I try not to get too upset when people come past me on the first day, but it still has an effect. There’s a negativity that sets in when people are faster than you. And it’s then you really need to focus and rationalize…I’m on my plan, I’m doing OK, ignore the other people. But it’s a very difficult one to just put totally aside–(P06)

#### Functional teams

Psychological benefits of teamwork. As the race progresses, the appeal of teaming up with other racers appears to intensify. While racers reported they did not experience loneliness, some racers did suggest that there were benefits to having company during the race. Teaming up with others was seen as a natural process which typically resulted in new friendships. Running with other racers was also viewed as a welcome distraction that staved off boredom and promoted time dilation. Some participants also openly admitted that they prefer the company of others when racing. At first glance this tactic of teaming up appears to clash with the overarching goal of “running my own race”. However, this conflict is less likely to occur in small teams where complimentary alliances are formed and clear expectations are set. In such cases, the social and psychological benefits of group membership are considered to outweigh the potential costs:

I tend to end up running with other people or other people are attracted to running with me. I think people quite like my company on the trail. I’ve kind of got a right balance of getting on with the job but having a bit of chitter chatter and a bit of a laugh at the same time—(P12)

No respondents said they suffered from loneliness during the race. However, loneliness was a concern for one participant who had watched a video blog of a racer who had suffered from loneliness on a previous Spine race. Another participant observed that while he did not experience loneliness, he did appreciate making human contact after prolonged periods of solitude during the race. Some racers proactively sought out the company of fellow racers early in the race or even in advance of the race. There is evidence to suggest that some racers even formed teams before the race began. Interestingly, it seems these pre-race partnerships or teams are often not formalized or verbalized in advance of a race. These tacit agreements between racers often remained unspoken and implicit.

Division of labor. There is evidence to suggest that race teams are formed even before the race begins. The findings of the study also suggest that being in a group lessens the opportunities for introspection and negativity. Participants also reported that being in a group provided them with emotional and motivational support as exemplified by the comments of P10. Conversely, the negative effects of being isolated from a group are demonstrated by the comments of P04:

Yes, I certainly helped XXXX at times. On a previous race, towards the end, I helped a guy who was there. He wasn’t looking very well, so he needed some motivation. You build the relationship very quickly…not necessarily on purpose, or by intention but just by the nature of the circumstance—(P10)

You tend to create demons in your own mind when you’re on your own…the mental breakdown started then. I was totally on my own on the second day, which may have contributed to me pulling out. I only did four hours on the second day—(P04)

Problem solving options and shared goals. In addition to providing emotional and motivational support, group members can help each other as unexpected problems arise. These helping behaviors are evidence of teamwork. Thus, groups of racers soon become functional teams. This suggests there can be high levels of teamwork and solidarity exhibited among racers. For racers at the back of the field (i.e., Back markers), the primary goal is to “break the Spine”. This goal of finishing the race under the time allowed is sufficiently challenging in itself for these athletes. Given that two thirds of racers fail to complete the race, the goal of simply finishing the race is the main motivation for many racers. The overarching goal to finish the race is the driving force that sustains forward momentum among back marker athletes. Importantly, the goal of completing the race informs subordinate goals that are often negotiated amongst team members. In order to stay as a unit, team members sometimes have to negotiate team goals that deviate from their own personal preferences (e.g., a negotiation of 3 hours of sleep and not 4 hours). If the cost of complying with the wishes of other team members does not incur a high personal cost for the racer, then overall team unity is likely to be increased overall. However, other aspects also influence the team’s cohesion. Participants reported that working as a team fostered a strong sense of “esprit de corps” and interdependency P06:

Yes [I was helping them], but I was [also] feeding off them [emotionally] as well, which was great…we’d set ourselves kind of a target to get there as soon as possible and we kept the momentum going. One of us would lead for a while and then we’d swap over. We’d work together, help each other and that’s what we did. It was a really good team spirit—(P06)

The strong “esprit de corps” within teams appears to prompt many racers to cross the finish line as a group. Historical data of Spine race results shows that racers often cross the line together, typically in groups of two or three racers. In the current study, 10 of the 22 finishers undertaking the Spine Fusion 2018 crossed the finish line with a fellow racer. In short, almost half (45%) of the race finishers were part of a team or a partnership. This high rate of co-operation between racers on the Spine race is a discernible pattern. The Spine race results from 2012–2018 suggest that, on average, almost most half (48%) of the racers who crossed the finish line, did so with another racer.

Further evidence of a team-based approach to racing is provided by participants in the current study. The Spine race finish traditionally involves racers touching the cornerstone of the pub at Kirk Yetholm. This ritual has an emotional significance for racers who view this as the end of the journey and assures them the coveted finisher’s medal. Anecdotal evidence provided by P06 recounts the team’s decision to cross the race finish line together.

I said, you know, we’ll stop a foot short of the wall and one, two, three and then place our hands together…we’d agreed it (finishing as a team). . . . . .we’d agreed it was a good thing to do—(P06)

Team members rarely break rank and cross the line ahead of their teammates as evidenced in the historical race results data. It appears the social pressure of the group ensures conformity. As the finish line approaches, team cohesion appears to strengthen, forged by the weight of shared experience.

#### Dysfunctional teams

Conflict: team goals vs. individual goals. When race plans differ significantly between team members, subordinate goals also become misaligned. In such instances, competing goals can become a source of conflict and these conflicts can be exasperated by fatigue and other situational stressors. Being on a dysfunctional team can lead to frustration and hostility within the group as evidenced by the comments of P12 who describes how one such situation escalated:

It became apparent that XXX strategy was incompatible with mine and XXXXX. He was trying to push his strategy [longer sleeps] on us without discussion. This caused friction. Additionally, XXX conversation style was stopping free conversation between XXXXX and I, which I resented. Also, he was at the back and not adding much to the team effort—(P12)

As frustration grows within a dysfunctional team, team members can adopt a number of different strategies to register their discontent (e.g. silence, unresponsive behaviors or even active avoidance of the individual). In order to remedy the situation, a team may surreptitiously try to “drop” a group member. The failure of the team to “drop” a member resulted in frustration. However, such conflict situations appear to be rare occurrences and participants typically reported they had very positive experiences within groups on ultra-races. Teaming up with other racers appears to be a widely used strategy among mid-ranking and back marker racers who support each other during the race.

### Race officials

This major dimension contains different themes: Influence of medics and influence of race volunteers.

#### Influence of medics

Social bonds through multiple interaction. The medics who volunteer to support the race can be considered part of the “Spine family”. The Spine family encompasses all those involved in the Spine race; the racers, the volunteers and the medics who give of their time to support the race. Due to the length of the course (410km), racers and medics meet multiple times. Unlike short ultra-races, multi day races like the Spine race result in repeated exposure to the same support staff. These repeat encounters appear to create social bonds between the racers and the medics. Indeed, one medic was observed shedding tears at the sight of one particular racer crossing the finish line. This emotional attachment is perhaps symptomatic of the escalating commitment felt between medics and the racers.

On this race these people (medics) travel with you through the journey. I saw XXXXX and XXXXX the medics, I can’t even remember how many times. They kept popping up every single time there was a medic, there was XXXXX and XXXXX there to see me.–(P09)

Fear of medics exerting expert power. Participants in the current study reported that the vast majority of their encounters with the race medics were very positive. However, participants did report some minor incidents which caused disagreement. The source of the disagreement was based on the perceived conflicting goals between the racers and the medics. A racer’s overarching goal of completing the race did not always align with the goals of the medics who had a duty of care to ensure the racer’s safety. This occasionally led to frustration. For example, one participant who was diabetic was encouraged to keep eating despite actively monitoring his own blood sugar levels and already feeling quite full. Another racer reported that his goal of carrying a light rucksack conflicted with the safety goal of the medic to ensure he carried enough water. The medics were highly regarded by all the participants in the current study. However, one participant reported that he was careful when making a phone call to the race HQ to query a medical issue he was experiencing. He reported that he did not want to raise the concerns of the medics as this might jeopardize his future participation in the race.

I tried to make it as neutral sounding as I could to stop the alarm bells ringing–(P08)

A racer in a different team expressed similar concerns and elected not to disclose his injury to medics. He also encouraged one of the participants not to divulge his own injury to medics. These admissions by racers suggest that goal focused racers can be distrustful of medics who wield the power to halt their race on medical grounds. The comments indicate that the power differential between the groups can be a source of concern, especially when the outcome of a medical prognosis is unknown:

And XXXX said, don’t mention it to the aid station people whatever you do, otherwise, they might stop you, they might stop us. And XXXX had some other issues with his eyes and he was like, I’m not telling them, don’t tell them, don’t tell them anything, they’ll pull us out of the race–(P12)

#### Influence of race volunteers

Mutual respect and a pay-it-forward ethos. A pay-it-back ethos appears to be commonplace in ultra-running circles. The relatively small number of athletes who undertake the Spine race ensures that close relationships are formed among fellow Spinners. Racers and those involved in supporting the race all share a mutual respect for each other. They are all members of the “Spine family”. The Spine race was first run in 2012 with just three entrants. In the intervening years, the notoriety of the race has fueled its popularity and engendered a strong sense of community among those involved in the race. This close-knit community is commonly referred to as “the Spine family”. Racers who complete the Spine race are frequently found to volunteer for marshaling duties in future races to support other aspirant racers.

It’s the people, it’s the way that the Spine family, as it’s called, has grown over the years … a group of people, that you’re all likeminded….you’re all up for a similar challenge—(P06)

There’s definitely this kind of…” we’re going to help you do it”, and then next year, “you can help us do it”, because it is a monumental challenge to finish any of the races really. So there is that kind of…we can do it together by supporting each other in alternate years, kind of feel to it. I hadn’t realized how much of a community it was–(P12)

Facebook group. The Spine family is maintained and nurtured using a closed Facebook group where racers interact freely with each other to share tips and advice on all aspects of the race. The social (media) exchanges on the Facebook Group revolve around the communal goal of completing the race.

I am part of a Facebook group and I feel supported–(P08)

### Family, friends & followers

This major dimension contains different themes: direct support and indirect influence.

In addition to the support that racers received from those directly involved in the race, racers also receive psychological support from friends and family. The friends and family of racers were able to constantly monitor the progress of racers using an online tracking website. Racers themselves were also able to use this website.

#### Direct support

Family members’ increasing emotional stability. Some racers reported that they availed of direct psychological support (phone call) to help them through the race. Indeed, some racers actively kept in touch with close family members and found that this helped them to overcome challenges. Making contact with close family members helped them to gain some perspective when they were experiencing difficulties.

She (the racer’s partner) is quite a positive reinforcement. I think it’s a source of normality, if you like. It brings me back from where I might be to where I really am–(P03)

Friends and family are typically a source of psychological support. Making contact with a close family member can allow a racer to discuss their difficulties and unburden themselves.

#### Indirect influence

Family members’ undermining emotional stability. Friends and family can undermine a racer’s chances of finishing a race even before it has begun. One participant noted that friends and family are likely to focus on the negative aspects of the race experience when they discuss the event with the athlete in advance of the race. These conversations often served to increase the athletes pre-race nerves. Some participants also reported that family members had the potential to inadvertently undermine their emotional stability at crucial times during the race. Indeed, some participants reported that they tried to actively protect themselves against the potentially subversive influence of family members. Racers adopted different approaches to achieve this. For example, some participants elected to psychologically distance themselves from friends and family by severing communications with them for the duration of the race. In other cases, racers attempted to regulate the nature of the interaction between family members. For example, one participant instructed his wife not to entertain any arguments he might make if he rang her while feeling low during the race:

I don’t keep in touch with my wife or my children but if I do, my wife’s got very specific instructions. If I phone her to tell her how bad a time I’m having and moaning about it, I’m not allowed to give in. So she knows that she’s not allowed to sympathize with me—(P02)

The dangers of contacting family members was also mentioned by another participant who recounted his memory of ringing home while feeling low during a different ultra-race. His comments suggest that ringing home is sometimes a dangerous tactic as it can stir up unhelpful emotions that may weaken the resolve of the racer. The sympathetic ear of a loved one can allow the racer “off the hook”. A concerned family member can validate the racers appraisal of the situation in an effort to comfort them. However, such an action may give the racer a license to quit if they are feeling particularly low at time:

In retrospect I think that I was ringing to validate my own quitting. I was saying this is how bad I feel—(P08)

Support via social media. In addition to family members, racers can solicit psychological support from a broad base of friends and digital followers. Followers could monitor the progress of racers using the online tracking website and then send messages of support via digital platforms such as WhatsApp and Facebook. One participant mentioned that he found it reassuring that his friends could track his progress online:

I knew the people who cared and who had done adventure sport would be watching. But that didn’t add any extra pressure, if anything, it added a bit of a safety net, I think–(P12)

Some participants were conscious of their online audience and even recorded podcasts or live Facebook videos to engage with their friends and followers. One participant wanted to share the joy of crossing the line with his followers, so he broadcast the event live on Facebook:

I had been interacting with so many people via Facebook who had kept me going and kept me moving I wanted them to be there when I crossed the line as well. I did my Facebook live post… I ran across the line and I did that whole thing live. I filmed myself coming in and it was awesome–(P09)

While online followers can be a source of support and motivation, some racers are less positively disposed to being tracked online. Indeed, a participant admitted that he felt uncomfortable about being tracked online by a mass online audience who could question his progress and make judgements on his race decisions. This social pressure is further amplified if a racer has undertaken charity fundraising and solicited money from members of the public:

It’s a pretty difficult, lonely experience, and it can be quite awful. I was ready to drop out on that occasion …then thought, but hang on, …you’ve just done this big thing for charity… so the money’s already there, and you’re going to embarrass yourself by giving up within the first few hours and it’s not going to look good—(P10)

The above comments again demonstrate the social nature of ultra-marathons. Modern technologies have empowered both athletes and audiences to fully immerse themselves in the social aspects of these sporting events. In addition to friends and family, participants also recounted favorable social interactions with members of the public. These interactions were often brief, but they served a number of positive functions.

### The general public

In this section, evidence of direct support and indirect pressure from the general public is presented.

#### Direct support

Encouragement. Participants generally reported positive interactions with members of the public. All participants reported that they interacted with the public as long as their race speed was not being unduly compromised. A number of racers commented that they enjoyed engaging with the general public as it lifted their mood as exemplified by the comments of P07.

I find that positive energy is reflected back at you. So if you need lifting or if you’re out there running, if someone smiles at you it lifts your mood and if your moods lifts you go faster. So if you go into a checkpoint and you’re absolutely miserable and you stomp around and you piss everyone off, they’re miserable back at you and your mood goes down whereas if you go in there smiling and you’re happy they will smile back at you. You can sort of thrive on that energy I found—(P07)

Racers also found that engaging with members of the public relieves the incessant internal monologue used to assess their goals and their race progress. Some participants reported they experienced a swell of pride when they encountered the public and explained the race challenge they were undertaking. These interactions served to increase racers’ self-esteem and often resulted in offers of food or navigational advice. In general, participants reported that members of the public were eager to provide support and lend assistance. Some members of the public were especially enthusiastic about the race. These “Spine groupies” actively monitored runners online so that they could make timely interventions when the racers passed their location on the course. Front-running athletes are especially likely to benefit from the kindness of strangers who were impressed with their running performance. However, all participants have the potential to benefit from encounters with the general public.

#### Indirect pressure

Resourceless back markers. Participants generally had very positive interactions with members of the public. However, racers at the back of the field reported that they don’t receive the same public praise and adulation bestowed upon the front running athletes. They racers can also miss on out some of the opportunities that can be availed of by the front running athletes:

By the time I got there, the barbecue had packed up and gone. When I arrived, the publican said, take your muddy boots off because all the rest have been in here with muddy boots and we’re just tidying up—(P03)

Misinformation. Occasionally, interactions with the public can lead to unforeseen problems. For example, runners can (un)intentionally be given misinformation by members of the public:

This farmer spotted me and he said, are you going north or south? So I said, I’m going north. So he said, well you see where that gate is over there, you need to be going through there, and I knew that was to the south. So I had to ignore what he was saying–(P10)

## Discussion

This research undermines the commonly held perception that ultra-running is a solitary sport. Indeed, the current research suggests that ultra-runners leverage many different relationships to help them overcome the challenges they encounter. Specifically, the racer’s social interactions with race officials; family/friends/followers; the general public and fellow racers are found to be important determinants of the athlete’s race experience. Interestingly, social interactions are found to occur before, during and after the race in both online and offline environments. Social interactions are especially important during the race when athletes are psychologically vulnerable.

Race situational stressors (e.g., sleep deprivation) undermine a racer’s ability to be wholly self-reliant. Many of the challenges faced on long ultra-races often have psychological components (e.g., motivation, problem solving) [41], that appear to be amenable to social solutions. It is therefore unsurprising that the majority of social interactions reported in the current study were found to support the athlete in their race. When a person integrates into a group, the relationships formed often support their personal needs and objectives. Research by [42, 43] suggests there are two different dimensions in social relationships, the instrumental-oriented dimension (i.e., instrumental social dimension) and the affective-oriented dimension (i.e., affective social dimension). The instrumental social dimension relates to all the task-oriented reasons why people stick together (i.e., common objectives, problem solving). In contrast, the affective social dimension relates to all the social and motivational supports that people gain by sticking together. These instrumental and affective social dimensions were inspired by research that sought to measure cohesiveness within sports teams [42, 43].

### Instrumental social dimension

Ultra-racers seem to strongly influence the race experience of their fellow runners. In the current study, racers directly influenced each other during the pre-race phase using the Official Spine Facebook group. Spinners shared tips and advice on all aspects of the race through this social media platform. This was an invaluable source of information for new racers. Advice received on the Facebook group influenced athlete’s decision-making and planning in advance of the race. The lively discourse and collaborative atmosphere on the Facebook group also helped to break down the barriers between racers. This collaborative ethos was also in evidence during the race. Racers were found to seek the support of fellow racers in times of difficulty during the race. Frequently, racers teamed up to overcome problems and support each other.

The potential advantages of joining a team appear to grow as the race progresses. As the challenges become greater, the costs associated with team membership become differentially less. Therefore, it is unsurprising that athletes’ group together to form teams. However, within the context of a race, this practice seems contrary to normal race goals. The prevalence of this pattern of behavior is surprising as runners are presumed to compete directly against each other. However, race data suggests that teamwork on the Spine race is not the exception, but the rule.

For front running racers, the potential for teamwork is less. Indeed, it seems self-evident that social interactions with fellow racers during competitive racing should negatively influence the athlete’s race experience. Furthermore, pre-race nerves can be exaggerated as a consequence of being surrounded by other nervous racers and not having a common instrumental objective (e.g., to win the race). Racers reported enacting avoidance strategies to counteract social pressures and pre-race nerves. Pre-race nerves also prompted racers to ruminate on their running kit choices and to engage in unhelpful upward social comparisons that served to heighten their pre-race nerves. Interestingly, during the early stages of the race, all athletes reported they were focused on pursing their own race goals. Consequently, few racers engaged with each other in any meaningful way during the early sections of the race. However, as the race progressed, situational stressors increased athlete’s willingness to interact with other racers. These social interactions were initially used to provide a distraction and pass the time. However, as the race progressed, complimentary alliances were forged between racers. “Teaming up” with other athletes’ often resulted in strong group cohesion that provided both motivational and psychological support to racers.

Group cohesion or cohesion within a team is widely studied in sport science [44]. It can be defined as “a dynamic process, which is reflected in the tendency for a group to stick together and remain united in the pursuit of its instrumental objectives and/or for the satisfaction of member affective needs” ([42], p. 213). In the current study, the competing and complimentary goals of team members had the potential to create conflict and co-operation among racers. Research suggests that the motivational climate within a team can determine the team cohesion. This is found to be especially true for ultra-race events that are both physically and psychologically demanding [45, 46, 47]. In the current study, participants reported that teaming up seemed to protect them from negative mood. Within a team, athletes were also more attentive to shared goals and had less time to internalize their own negative thoughts and feelings. The company of others also served as a welcome distraction that helped to relieve boredom and desensitized them to the passage of time. The group dynamics also relived racers of constantly assessing their own individual speed and progress, thereby overthrowing “the tyranny of the (GPS) watch”. However, differing personality types and situational stressors exaggerated both favorable and unfavorable social influences.

Past research has already shown that inter-personal factors and intra-personal factors influence group formation and group cohesion (for a review see [29]). Research also finds that clear communication and problem-solving behaviors characterize successful teams [36]. Team cohesion is found to occur when subordinate racing goals are shared by group members. In the current study, the shared goals of Spinners allowed for the effective division of labor among team members. These findings are supported by [26] who explored the effects of harmonious and obsessive passions on team performance. It is worth noting that team cohesion has two dimensions; task cohesion and social cohesion. Task cohesion team goals [48, 49, 50] relate to the ability of the group to work towards a shared goal or performance objective. Shared task goals have the potential to unite a group and induced a strong “esprit de corps” among team members. Team cohesion is also dependent upon the social cohesion within the group. Social cohesion relates to the ability of the group to psychologically support each other during the goal pursuit process [48, 49]. A willingness to share goals and their associated workload is also an important part of establishing a place in a team. Where personalities clash or where goals and workloads are not shared, there is the potential for conflict. Nevertheless, there is little doubt of a tendency towards teamwork on the Spine race. This teamwork is evidenced despite the resurgence of competitive goals towards the end of the race. The lead author once heard a Spinner say that the “real race doesn’t start until about 30 miles from the end”. While this may be true, this competitive pressure does not seem to be strong enough to fragment teams during the final stages of the race. Race statistics suggest that team formation was very common on the Spine race. The race results from 2012–2018 suggest that, on average, almost most half (48%) of the racers who crossed the Spine race finish line, did so with another racer. This statistic is striking. It suggests that the race towards the finish line tends to be undertaken by teams rather than individuals. This trend is particularly visible among mid-pack and back-marker runners.

### Affective social dimension

The Spine family is the close-knit community of racers and volunteers who are actively involved or historically linked to the Spine race. The Spine Facebook group is thus the home of the Spine family. However, in addition to racers past and present, the Spine family is also composed of race officials and race medics. While medics are primarily focused on providing medical support, the current research suggests that there is an important social and psychological aspect to their role. Over the course of the seven days, medics may be based at up to 10 different locations along the race route. The duration of the race and the relatively small number of racers and medics allows for multiple interactions between these two groups. In addition, medics move up along the race route in tandem with the racers as the race progresses. This increases the likelihood that a racer and a medic will encounter each other multiple times, thus allowing a race relationship to form.

This research suggests that the relationships between racers and medics is typically positive and based upon mutual respect. Racers reported they enjoyed meeting the medics and appreciated their efforts to keep them in the race. Racers were mindful that their invaluable medical assistance supported their bid to finish the race. Medics, for their part, were found to express their admiration of the athletes and some even wrote motivational messages on the foot dressings of the athlete’s they had treated. The lead author also observed one of the medics become emotional as a racer she has repeatedly encountered finally crossed the finish line. Fighting back tears, the medic appeared delighted to have the opportunity to present the finishers medal to the athlete.

None of the participants in the current study reported negative social interactions with medics. However, a number of participants did report that they were reluctant to provide medics with a fully transparent account of their state. They reported they were fearful that medics might invoke their right to remove them from the race if the medical condition was deemed too serious. In situations where there was a divergence between the safety goals of the medics and the race goals of the athletes, the athletes tried to ensure that their respective goals were not diametrically opposed to those of the medics by using temperate language. In some cases, medics were duty bound to insist that racers adopt certain risk reduction strategies (e.g. carrying more water) despite the mild protestations of athletes (e.g., who were adamant they needed to travel light). Despite these minor issues, the study revealed that strong relationships were formed between racers and medics during the race.

Friends and family were also found to offer important psychological support to the athletes. These “dot watching” groups tracked the athletes progress online through the race website and often sent the athlete supportive messages using Facebook or WhatsApp. Racers took different views with regard to being tracked online. Many racers thought it was beneficial to have friends and family actively monitor their race progress. This use of new technologies has become a way of highlighting an individual’s social affinity to a group [51]. However, some athletes reported that social media and race tracking technologies can exert unwanted social pressure.

The Spine race now has a strong social media presence and generates a lot of attention among different groups: members of the public (followers), friends and family. Some participants suggested that the social pressure of being observed made them wonder how people would interpret their race decisions. This pressure was especially salient for athletes who had done charity fundraising in advance of the race. The fact that members of the public had donated money increased the social pressure felt by racers. Racers who were not fund raising were less likely to feel social pressure.

Some racers actively embraced social media and reported that their online social interactions increased their enjoyment of the race. For example, some participants recorded podcasts in order to share the race experience with others. One racer even broadcasted video updates of his race via Facebook updates. This racer mentioned that he even slowed down at the very end of the race so that he could share his early morning race finish with as many friends as possible. He reported that he wanted to pay back his followers for supporting him throughout the race. All of these behaviors suggest there is a strong social component to ultra-running. It also suggests that the social aspects of ultra-running have grown in recent years with the advent of new technologies and social media. Participants reported that this indirect psychological support had a positive effect on their race experience.

Some participants reported that they benefited from accessing direct psychological and emotional support. This result is in line with the findings of past research [52]. In the current study, some participants solicited psychological support from close family members by phoning them. However, other racers intentionally elected to forego contact with close family members. For example, some racers reasoned that talking to their children might stir up unhelpful emotions that might direct their focus away from the race. Contact with non-family members (i.e., the general public) was similarly dichotomous in potential outcomes.

Racers generally reported they had very positive experiences with members of the general public. Indeed, the racers frequently reported that they were held in high regard by those they encountered along the route. Racers typically benefited from their interactions with the general public and some racers reported they experienced a motivational boost when they explained the challenges they were undertaking to a suitably impressed audience. Racers also felt a positive motivational effect when they speed past members of the public on the route. This again instilled a sense of achievement and reinforced their perception of being special.

Occasionally, social comparisons with the public had negative effects on athletes’ race experience. Comparing themselves to members of the public who were blissfully unaware of their pain and discomfort made one participant feel resentful of their carefree indifference. Interactions with the public can also be problematic if misinformation (e.g. incorrect directions) is provided by members of the public. Nevertheless, interacting with members of the general public was usually seen as a welcome distraction from the race. Racers reported that members of the public frequently offered them food and water in addition to psychological support and motivation. However, one back marker athlete observed that front running athletes were the chief beneficiaries of public support. Athletes at the back of the course were less likely to receive the same level of attention as those positioned at the front of the pack.

The present study has a number of limitations. For example, the current study did not investigate if the social and interpersonal aspects of the race differed among those who finished the race and those who withdrew from the race. It would be interesting to compare and contrast the differences in the social experiences of these two cohorts. Future research could investigate if the type and valance of social interactions experienced during the race can determine downstream variables such as race outcomes.

Another limitation of the current research is the uniform gender of participants. Only male athletes volunteered to participate in the current study. This is likely to be an artifact of the Spinner racer demographic. Few female athletes partake in the Spine race. The gender bias in the current study limits the generalizability of the findings. Future research should seek to include female athletes in order to assess if there are differences between the social experiences of male and female racers. Another limitation of the current study is the retrospective nature of the data collection. Inherent in this retrospective study design are the risks and challenges associated with memory recall. The process of building new meaning requires that runners explain their histories from compromised or incomplete recollections. Importantly, studies have confirmed that it is possible to limit the weaknesses of traditional verbal reporting [5]. The current study sought to minimize these weaknesses by collecting data using the modified version of the DRM. While no methodology is infallible, this approach was particularly helpful in documenting and understanding the chronology of unfolding event. Nevertheless, future studies could potentially incorporate additional data collection techniques that might help with the triangulation of the data. Adopting new and innovative methodological approaches (e.g. observation) could potentially increase the ecological validity of future studies while supporting the phenomenological aspects of the research.

### Perspectives

The current research provides new insights into the psychology of ultra-running. Until now, the positive and negative social influences that affect ultra-racers have been largely overlooked in the literature. The current research sought to address this conspicuous gap in the literature. The results suggest that there is a strong social dimension to ultra-running. Interestingly, the vast majority of social interactions reported by runners have a positive effect on race experience and often led to interpersonal relationships. However, some social interactions can generate negative feelings and these scenarios need to be considered by future athletes (e.g. peer pressure from online and offline sources and goal conflicts with fellow racers, medics and the general public).

Future research could explore the social aspects of ultra-running from different points of view, for example from female athletes or elite/experienced and non-elite/inexperienced athletes. Future research could also profitably explore the impact of new technologies on athlete’s race experience. Social media and digital communications have infiltrated all aspects of modern life and have ushered in an age of unprecedented inter-connectivity. Future research should explore how these new technologies shape social interactions and interpersonal relationships within the context of endurance sports. The current research suggests that athletes need to extend their race preparation beyond the physical and mental training they currently engage in. Ultra-athletes should be cognizant of the social pressures that manifest themselves within the race situation. Preparing for the social aspects of ultra-running has the potential to dramatically improve their future race experiences.

## Conclusions

Fellow racers, volunteers, medics, friends and family and even the general public can provide positive psychological support for runners. These cohorts seem to play an important role in overcoming the challenges that ultra-racers experience. The current research dispels the myth that ultra-running is a solitary sport. Indeed, the findings suggest that the social aspects of ultra-running play an integral role in shaping an athlete’s race experience. Future research should continue to investigate the social aspects of ultra-racing that, to date, have remained largely under reported.

## Supporting information

S1 Appendix Pre-race questionnaire. (DOC)

S2 Appendix Day Reconstruction Method (DRM). (DOC)

S3 Appendix Post-race interview topic guide. (DOCX)
