# Supplementary material for: From social interactions to interpersonal relationships: Influences on ultra-runners’ race experience
Source: PLoS One. 2019 Dec 2;14(12):e0225195. doi: 10.1371/journal.pone.0225195 (PMC6886831; doi:10.1371/journal.pone.0225195)
Supplement: S1 Appendix — (DOC) [file pone.0225195.s001.doc]

**Survey 1**

**First we have some general questions about your fitness training and sporting background.**

Name ______________

What is your gender? _____________

Age ____________years

Profession ____________

Sporting background and skill set

Please give us an idea of your sporting background and skill set. E.g. previous races, orienteering experience, hill walking experience, mountaineering experience, adventure racing experience. Please also include any other information you feel may be relevant e.g. qualifications, training etc (e.g. Mountain Leader qualification, military experience (branch/time served) etc.)

**IN THIS SURVEY, WHERE YOU SEE LINES TO INDICATE A TEXT RESPONSE (E.G. SEE SECTION BELOW) PLEASE SIMPLY DELETE THE LINES AND TYPE IN YOUR RESPONSE. PLEASE PROVIDE AS MUCH DETAIL AS YOU CAN.** ______________________________________________________________________

______________________________________________________________________

______________________________________________________________________

______________________________________________________________________

______________________________________________________________________

______________________________________________________________________

______________________________________________________________________

______________________________________________________________________

______________________________________________________________________

______________________________________________________________________

______________________________________________________________________

______________________________________________________________________

______________________________________________________________________

______________________________________________________________________

______________________________________________________________________

______________________________________________________________________

______________________________________________________________________

______________________________________________________________________

______________________________________________________________________

______________________________________________________________________

General sports participation

What other sports/outdoor/fitness activities do you engage in?

____________________________________________________________________________________________________________________________________________________________________________________________________________________________________________________________________________________________________________________________________________________________________________________________________________________________________________________________________________________________________________________________________________________________________________________________________________________________________________________________________________________________________________________________

Overall, how many hour a week do you spend exercising? __________.hours

Running training

How long do you been trail running? _____________________

How many ultra-marathons have you completed (approx) _____________________

How many races do you in a year (approx) _____________________

How many hours running training per week do you do? _____________________

Do you train alone or in a club? _____________________

Please give an indication of your typical training week preparing for this race (e.g. hours and kms/miles run, gym work etc).

______________________________________________________________________

______________________________________________________________________

______________________________________________________________________

______________________________________________________________________

______________________________________________________________________

______________________________________________________________________

______________________________________________________________________

______________________________________________________________________

______________________________________________________________________

______________________________________________________________________

______________________________________________________________________

What motivated you to sign up for this race?

______________________________________________________________________

______________________________________________________________________

______________________________________________________________________

______________________________________________________________________

______________________________________________________________________

______________________________________________________________________

______________________________________________________________________

______________________________________________________________________

______________________________________________________________________

______________________________________________________________________

______________________________________________________________________

Have you participated in the Spine race before (Y/N)? ________

If yes please indicate the race/year/result below

______________________________________________________________________

Race Year(s)+ Completed race/ did not finish (DNF)___

E.g. (2017 completed), 2016 (DNF)

Spine Race Fusion _____________________________________

Spine Race Flare _____________________________________

Spine Race Challenger _____________________________________

Spine Race (Winter) _____________________________________

What aspect of your race are you most confident about? E.g. Your fitness/running ability, your navigational ability, your will power, your gear, your race-plan, your sleep strategy, (other? please specify). Please explain why you are confident about this aspect of your race.

______________________________________________________________________

______________________________________________________________________

______________________________________________________________________

______________________________________________________________________

______________________________________________________________________

______________________________________________________________________

______________________________________________________________________

______________________________________________________________________

______________________________________________________________________

______________________________________________________________________

______________________________________________________________________

______________________________________________________________________

______________________________________________________________________

______________________________________________________________________

______________________________________________________________________

______________________________________________________________________

______________________________________________________________________

______________________________________________________________________

______________________________________________________________________

______________________________________________________________________

______________________________________________________________________

______________________________________________________________________

What aspect of your race are you least confident about? E.g. Your fitness, your navigational ability, your will power, your gear, your race plan, your sleep strategy (other? please specify). Please explain why you are not confident about this aspect of the race.

______________________________________________________________________

______________________________________________________________________

______________________________________________________________________

______________________________________________________________________

______________________________________________________________________

______________________________________________________________________

______________________________________________________________________

______________________________________________________________________

______________________________________________________________________

______________________________________________________________________

______________________________________________________________________

______________________________________________________________________

______________________________________________________________________

______________________________________________________________________

______________________________________________________________________

______________________________________________________________________

______________________________________________________________________

______________________________________________________________________

______________________________________________________________________

______________________________________________________________________

______________________________________________________________________

______________________________________________________________________

**Thank you!**
