# Supplementary material for: From social interactions to interpersonal relationships: Influences on ultra-runners’ race experience
Source: PLoS One. 2019 Dec 2;14(12):e0225195. doi: 10.1371/journal.pone.0225195 (PMC6886831; doi:10.1371/journal.pone.0225195)
Supplement: S2 Appendix — (DOC) [file pone.0225195.s002.doc]

**Survey 2**

**INSTRUCTIONS**

On the next pages, please describe your race. Think of your race as a continuous series of scenes or episodes in a film. For each section of the race, landmarks have been listed to help you recall the route and your actions during that time. These are just suggested time-points/episodes to help you remember. You may include episodes from different sections of the route if you wish. To complete the survey, please write down the approximate times at which each episode began and ended. Episodes that people identify usually last between 15 minutes and 2 hours. For example, the end of one episode might involve going to a different location, ending one activity and starting another or a change in the people you are interacting with. There is one page for each of the race sections (see below). Athletes competing in the Spine Flare race need to complete the first 3 sections (up to CP2 Hawes). Athletes competing in the Spine Fusion race need to complete all 7 sections (up to the finish at Kirk Yetholm).

Race sections

START - CP 1 (HEBDEN)

CP 1 - CP1.5 (MALHAM TARN)

CP1.5 - CP2 (Hardraw)

CP 2 - CP3 (MIDDLETON)

CP 3 - CP 4 (ALSTON)

CP 4 - CP 5 (BELLINGHAM)

CP 5 - FINISH (KIRK YETHOLM)

There is space to list 7 episodes for each race section (although you may not need that many). It is not necessary to fill up all of the spaces – breakdown your race in a way that makes sense to you. Try to recall each episode in detail and write a few words that will remind you of exactly what was going on. It is important to try to capture:

- what you were doing
- how you felt (i.e. your mood)
- your thoughts
- your interactions with other racers during that episode (if applicable).

**NOTE: What you write here in Survey 2 will not be seen by anybody else. What you write in this survey only has to make sense to you. This survey is simply to help you remember what actually happened during your race. The primary role of this survey is to allow you to gather your thoughts before completing Survey 3. Survey 2 is yours to keep if you wish.**

| **START - CP 1 (HEBDEN)** | | | | |
| --- | --- | --- | --- | --- |
|  |  |  | |  |
|  |  |  | |  |
| Episode name | Time period (e.g.)  00.00 – 02.00 | | **Your notes:**  **What happened? What were you doing? Mood? Feelings? Thoughts? Interactions with others?** | |
| 1. START |  |  |  |  |
|  | _______ | __________________________________________ | | |
| 2. Kinder Scout |  |  |  |  |
|  | _______ | __________________________________________ | | |
| 3. Snake Pass,  Bleaklow Head |  |  |  |  |
|  | _______ | __________________________________________ | | |
| 4. Torside  Reservoir,  Laddow Rocks |  |  |  |  |
|  | _______ | __________________________________________ | | |
| 5. Crowden,  Wessenden  Reservoir |  |  |  |  |
|  | _______ | __________________________________________ | | |
| 6. M62 Crossing |  |  |  |  |
|  | _______ | __________________________________________ | | |
| 7. White Horse  Pub, Stoodley  Pike, Hebden |  |  |  |  |
|  | _______ | __________________________________________ | | |

Your extra notes: __________________________________________________________________________________________________________________________________________________

| **HEDDEN (CP 1) - MALHAM TARN (CP 1.5)** | | | | |
| --- | --- | --- | --- | --- |
|  |  |  | |  |
| Episode name | Time period (e.g.)  00.00 – 02.00 | | **Your notes:**  **What happened? What were you doing? Mood? Feelings? Thoughts? Interactions with others?** | |
| 1. **Hebden (CP1),**  Colden |  |  |  |  |
|  | _______ | __________________________________________ | | |
| 2. Top Withins,  Bothy |  |  |  |  |
|  | _______ | __________________________________________ | | |
| 3. Ponden Reservoir |  |  |  |  |
|  | _______ | __________________________________________ | | |
| 4. Crowling |  |  |  |  |
|  | _______ | __________________________________________ | | |
| 5. Lothersdale |  |  |  |  |
|  | _______ | __________________________________________ | | |
| 6. Thomas-in-  Craven, East  Marton, Gargrave |  |  |  |  |
|  | _______ | __________________________________________ | | |
| 7. Airton, Hanlith,  Malham Cove  Malham Tarn |  |  |  |  |
|  | _______ | __________________________________________ | | |

Your extra notes: ___________________________________________________________________________________________________________________________________________________________________________________________________________________________

| **MALHAM TARN (CP 1.5) –HARDRAW (CP2)** | | | | |
| --- | --- | --- | --- | --- |
|  |  |  | |  |
| Episode name | Time period (e.g.)  00.00 – 02.00 | | **Your notes:**  **What happened? What were you doing? Mood? Feelings? Thoughts? Interactions with others?** | |
| 1. **Malham Tarn**  (**CP1.5)** |  |  |  |  |
|  | _______ | __________________________________________ | | |
| 2. Fountains Fell |  |  |  |  |
|  | _______ | __________________________________________ | | |
| 3. Pen-y-ghent |  |  |  |  |
|  | _______ | __________________________________________ | | |
| 4. Horton in  Ribblesdale |  |  |  |  |
|  | _______ | __________________________________________ | | |
| 5. Cam Road |  |  |  |  |
|  | _______ | __________________________________________ | | |
| 6. **Hardraw** **(CP 2)** |  |  |  |  |
|  | _______ | __________________________________________ | | |

Your extra notes: ________________________________________________________________________________________________________________________________________________________________________________________________________________________________________________________________________________________________________________________________________________________________________________________________________________________________________________________________________________________________________________________________________________________________________________________________________

| **HARDRAW (CP 2) –MIDDLETON (CP 3)** | | | | |
| --- | --- | --- | --- | --- |
|  |  |  | |  |
| Episode name | Time period (e.g.)  00.00 – 02.00 | | **Your notes:**  **What happened? What were you doing? Mood? Feelings? Thoughts? Interactions with others?** | |
| 1. **Hawes (CP 2)** |  |  |  |  |
|  | _______ | __________________________________________ | | |
| 2. Great Shunner  Fell |  |  |  |  |
|  | _______ | __________________________________________ | | |
| 3. Thwaite |  |  |  |  |
|  | _______ | ______ ___________________________________ | | |
| 4. Keld |  |  |  |  |
|  | _______ | __________________________________________ | | |
| 5. Tan Hill |  |  |  |  |
|  | _______ | __________________________________________ | | |
| 6. Sleightholme  Moor (after Tan  Hill), Harter Fell |  |  |  |  |
|  | _______ | __________________________________________ | | |
| 7. **Middleton**  **(CP3)** |  |  |  |  |
|  | _______ | __________________________________________ | | |

Your extra notes: ____________________________________________________________________________________________________________________________________________________________________________________________________________________________________________________________________________________________________

| **MIDDLETON (CP 3) – ALSTON (CP 4)** | | | | |
| --- | --- | --- | --- | --- |
|  |  |  | |  |
| Episode name | Time period (e.g.)  00.00 – 02.00 | | **Your notes:**  **What happened? What were you doing? Mood? Feelings? Thoughts? Interactions with others?** | |
| 1. **Middleton**  **(CP3)** |  |  |  |  |
|  | _______ | __________________________________________ | | |
| 2. Low Force  High Force |  |  |  |  |
|  | _______ | __________________________________________ | | |
| 3. Cauldron Snout,  High Nic Cup |  |  |  |  |
|  | _______ | __________________________________________ | | |
| 4. Dufton |  |  |  |  |
|  | _______ | __________________________________________ | | |
| 5. Cross Fell,  Greggs Hut |  |  |  |  |
|  | _______ | __________________________________________ | | |
| 6. Garrigill |  |  |  |  |
|  | _______ | __________________________________________ | | |
| 7. **Alston (CP4)** |  |  |  |  |
|  | _______ | __________________________________________ | | |

Your extra notes: ________________________________________________________________________________________________________________________________________________________________________________________________________________________________________________________________________________________________

| **ALSTON (CP 4) – BELLINGHAM (CP 5)** | | | | |
| --- | --- | --- | --- | --- |
|  |  |  | |  |
| Episode name | Time period (e.g.)  00.00 – 02.00 | | **Your notes:**  **What happened? What were you doing? Mood? Feelings? Thoughts? Interactions with others?** | |
| 1. **Alston (CP4)** |  |  |  |  |
|  | _______ | __________________________________________ | | |
| 2. Slaggyford |  |  |  |  |
|  | _______ | __________________________________________ | | |
| 3. Greenhead |  |  |  |  |
|  | _______ | __________________________________________ | | |
| 4. Steel Rigg,  Hadrian s Wall |  |  |  |  |
|  | _______ | __________________________________________ | | |
| 5. Forestry after  Hadrains wall, |  |  |  |  |
|  | _______ | __________________________________________ | | |
| 6. Shitlington Crags |  |  |  |  |
|  | _______ | __________________________________________ | | |
| 7. **Bellingham**  **(CP5)** |  |  |  |  |
|  | _______ | _________________________________________ | | |

Your extra notes:

____________________________________________________________________________________________________________________________________________

____________________________________________________________________________________________________________________________________________

|  |  |  |  |  |
| --- | --- | --- | --- | --- |
| **BELLINGHAM (CP 5) – KIRT YETHHOLM** | | | | |
|  |  |  | |  |
| Episode name | Time period (e.g.)  00.00 – 02.00 | | **Your notes:**  **What happened? What were you doing? Mood? Feelings? Thoughts? Interactions with others?** | |
| 1. **Bellingham**  **(CP5)** |  |  |  |  |
|  | _______ | __________________________________________ | | |
| 2. Padon Hill |  |  |  |  |
|  | _______ | __________________________________________ | | |
| 3. Kinder Forest |  |  |  |  |
|  | _______ | ______ ___________________________________ | | |
| 4. Byrness |  |  |  |  |
|  | _______ | __________________________________________ | | |
| 5. Hut 1 (Lamb Hill  Refuge Hut) |  |  |  |  |
|  | _______ | __________________________________________ | | |
| 6. Cheviot Ridge,  Hut 2 |  |  |  |  |
|  | _______ | __________________________________________ | | |
| 7. The Schil,  **Kirk Yetholm**  **(Finish)** |  |  |  |  |
|  | _______ | __________________________________________ | | |

Your extra notes:

____________________________________________________________________________________________________________________________________________

Note

Please look over your diary once more. Are there any other episodes that you’d like to revise or add more notes to? Is there an episode that you would want to break up into two parts? If so, please go back and make the necessary adjustments on your diary pages. If not, then please complete Survey 3 by referring to the notes you have made here.
