# Supplementary material for: From social interactions to interpersonal relationships: Influences on ultra-runners’ race experience
Source: PLoS One. 2019 Dec 2;14(12):e0225195. doi: 10.1371/journal.pone.0225195 (PMC6886831; doi:10.1371/journal.pone.0225195)
Supplement: S3 Appendix — (DOCX) [file pone.0225195.s003.docx]

Interview Topic Guide

| **SPINE FLARE** | **SPINE FUSION** |
| --- | --- |
| START - CP 1 (HEBDEN) | START - KINDER SCOUT |
| CP 1 - CP1.5 (MALHAM TARN) | SNAKE PASS |
| CP1.5 – CP2 (HAWES) | BLEAKLOW |
| CP2 – CP3 (MIDDLETON) | LADDOW ROCKS |
| CP3 - CP 4 (ALSTON) | M62 CROSSING |
| CP4-CP 5 (BELLINGHAM) | WHITE HORSE/ STOODLEY PIKE |
| CP 5 - FINISH (KIRT YETHOLM) |  |

**Action (A) questions.**

What were you doing?

What did you do when you encounter other people?

**Thoughts (T) questions.**

What did you think?

What were your thoughts when you encounter other people?

**Feelings (F) questions.**

What was your feeling at that moment?

What was your feeling when you encounter other people?
